# Association of early cleavage, morula compaction and blastocysts ploidy of IVF embryos cultured in a time-lapse system and biopsied for genetic test for aneuploidy

**DOI:** 10.1038/s41598-023-51087-z

**Published:** 2024-01-07

**Authors:** H. De Martin, T. C. S. Bonetti, C. A. Z. Nissel, A. P. Gomes, M. G. Fujii, P. A. A. Monteleone

**Affiliations:** 1 Centro de Reprodução Humana Monteleone, Rua Lima Barros, 61 Jardim Paulista, São Paulo, SP CEP 04503-030 Brazil; 2 Disciplina de Ginecologia-Departamento de Obstetrícia e Ginecologia, Faculdade de Medicina da Universidade de São Paulo, Av. Dr. Enéas Carvalho de Aguiar, 255-10 Andar-Cerqueira César, São Paulo, SP CEP 05403-000 Brazil; 3 Departamento de Ginecologia, Escola Paulista de Medicina - Universidade Federal de São Paulo, Rua Pedro de Toledo, 781. 4º andar. Vila Clementino, São Paulo, SP 04039030 Brazil

**Keywords:** Medical research, Outcomes research, Translational research

## Abstract

IVF embryos have historically been evaluated by morphological characteristics. The time-lapse system (TLS) has become a promising tool, providing an uninterrupted evaluation of morphological and dynamic parameters of embryo development. Furthermore, TLS sheds light on unknown phenomena such as direct cleavage and incomplete morula compaction. We retrospectively analyzed the morphology (Gardner Score) and morphokinetics (KIDScore) of 835 blastocysts grown in a TLS incubator (Embryoscope+), which were biopsied for preimplantation genetic testing for aneuploidy (PGT-A). Only the embryos that reached the blastocyst stage were included in this study and time-lapse videos were retrospectively reanalysed. According to the pattern of initial cleavages and morula compaction, the embryos were classified as: normal (NC) or abnormal (AC) cleavage, and fully (FCM) or partially compacted (PCM) morulae. No difference was found in early cleavage types or morula compaction patterns between female age groups (< 38, 38–40 and > 40 yo). Most of NC embryos resulted in FCM (≅ 60%), while no embryos with AC resulted in FCM. Aneuploidy rate of AC-PCM group did not differ from that of NC-FCM group in women < 38 yo, but aneuploidy was significantly higher in AC-PCM compared to NC-FCM of women > 40 yo. However, the quality of embryos was lower in AC-PCM blastocysts in women of all age ranges. Morphological and morphokinetic scores declined with increasing age, in the NC-PCM and AC-PCM groups, compared to the NC-FCM. Similar aneuploidy rates among NC-FCM and AC-PCM groups support the hypothesis that PCM in anomalous-cleaved embryos can represent a potential correction mechanism, even though lower morphological/morphokinetic scores are seen on AC-PCM. Therefore, both morphological and morphokinetic assessment should consider these embryonic development phenomena.

## Introduction

In IVF laboratories worldwide, embryos are mostly selected for transfer primarily based on their development rate and morphological characteristics. However, embryo viability is only weakly correlated with its microscopic appearance^1,2^. Furthermore, aneuploidies affect more than 50% of human IVF embryos, and the extent to which morphology is affected by aneuploidy remains unclear^3–6^. The use of preimplantation genetic testing for aneuploidy (PGT-A) prior to single embryo transfers has become a common clinical practice with the aim of trying to improve live birth rates per transfer and reducing miscarriages^7–9^. However, PGT-A is an invasive and labour-intensive technique that adds a significant cost to the IVF cycle^10–12^. Still, the transfer of euploid embryos is not cost-effective for most patients, except for advanced maternal age, recurrent miscarriages, and recurrent implantation failures^13,14^.

In recent years, the time-lapse system (TLS) has become a promising tool for improving embryo selection and providing an uninterrupted evaluation of morphological and dynamic parameters^15–17^. This innovative technology has revolutionized observation of embryo development, thus offering a more accurate quantification of cellular kinetics and cell cycle events than static morphological assessments^18^. In addition to accurate recording of well-known events, such as the first cleavage, it also sheds light on previously unknown phenomena, such as multipolar mitosis^19–23^. Using TLS technology, specific dysmorphisms such as multinucleation, direct uneven cleavage, reverse cleavage, and irregular chaotic division in human preimplantation embryos can be evaluated in more detail^10,24–26^.

The most common form of abnormal cleavage is Direct Uneven Cleavage (DUC), also known as tripolar mitosis, wherein a single blastomere splits directly from one cell to three or more cells^10,25,27–29^. The first studies analyzing TLS data described DUC as an indicator of poor prognosis^28,30^. When DUC occurs, the earlier the occurrence of it, the greater the damage to the embryo. If DUC occurs in the first mitotic division (DUC 1–3), it generates blastomeres with genomic loss^23,29^, and all blastomeres are affected. The embryo will likely not advance beyond the morula stage. However, when the first cleavage is normal and DUC occurs during the second mitotic division (DUC 2–5), affected blastomeres are formed; however, there are still unaffected blastomeres resulting from the first cleavage. Generally, affected blastomeres undergo developmental arrest and unaffected blastomeres can continue their development, giving rise to a morula and later to a blastocyst^22,23,31^. Another early cleavage anomaly is the precocious or Rapid Cleavage (RC), where the first mitotic division is followed by another mitotic division in less than 5 h in one of the blastomeres. This fast process does not allow enough time for chromosomal duplication and results in one normal blastomere and two blastomeres with altered genetic content. These two types of anomalous cleavage affect the chromosomal distribution in blastomeres and consequently their developmental potential^31^.

Another two points of interest are embryo compaction and morula formation. Those are complex processes occurring immediately before blastocyst formation when the embryo becomes capable of implantation, which has long been neglected. With the advent of the TLS, dynamic observation of these processes has become possible^3,32–34^. More recent studies showed the phenomenon of partially compacted morula (PCM), which is defined as those showing cells excluded from the compaction process from the outset or extruded from an already-compacted morula^35–37^.

TLS has been available for over 10 years and widely used in IVF clinics. In the current study, we have used this tool for complementary analysis of the embryos, other than those regularly performed by the machine. According to our observations of morphological and morphokinetic parameters generated by TLS, and in accordance with published recent literature, abnormalities observed in the first mitotic divisions of the embryo are associated with patterns of embryonic compaction and morula formation. Moreover, although these disorders results in worse morphological and morphokinetic scores of the generated blastocyst, they may not influence the ploidy of this blastocyst. To study this hypothesis, we reviewed TLS videos of blastocysts biopsied for PGT-A and analyzed the association between early cleavage anomalies, compaction disorders during morula formation, and genetic outcomes of the respective formed blastocysts.

## Results

This study evaluated 835 embryos from 195 ICSI cycles (189 patients) that reached the blastocyst stage and were biopsied for PGT-A. The female age ranged from 25 to 47 years (38.2 ± 3.5). Analyses on early cleavage and morula compaction patterns were performed for all embryos and stratified according to maternal age into < 38 (n = 314), 38–40 (n = 323), and > 40 (n = 198).

The most displayed condition regarding the early cleavages and morula compaction, were normal cleavage (NC) (1–2–4; n = 697, 83%), and full compacted morula (FCM) (n = 516, 62%), respectively. Table 1 shows the complete description of each subgroup according to early cleavage and compaction process, for the total of embryos and according to female age. No differences were observed in the frequencies of early cleavage and compaction stage alterations according to female age subgroups.

**Table 1:** Embryo classification based on early cleavage (A) and compaction at the morula stage (B), according to female age subgroups.

	< 38 years old	38–40 years old	> 40 years old	Total	P
A: classification of zygote initial cleavage					
Normal Cleavage (NC; 1–2-4)	262 (83%)	263 (81%)	172 (87%)	697 (83%)	0.298
Rapid Cleavage (RC; 1–2-3)	29 (9%)	27 (8%)	10 (5%)	66 (8%)	
Direct Uneven Cleavage (DUC-2; 1–2-5)	23 (7%)	33 (10%)	16 (8%)	72 (9%)	
Total	314	323	198	835	
B: patterns of compaction at the morula stage					
Fully compacted morula (FCM)	204 (65%)	200 (62%)	112 (57%)	516 (62%)	0.144
Partial compacted morula with excluded cells (Exc-PCM)	76 (24%)	79 (24%)	49 (25%)	204 (24%)	
Partially compacted morula with extruded cells (Ext-PCM)	34 (11%)	44 (14%)	37 (19%)	115 (14%)	
Total	314	323	198	835	

**Figure 1:**
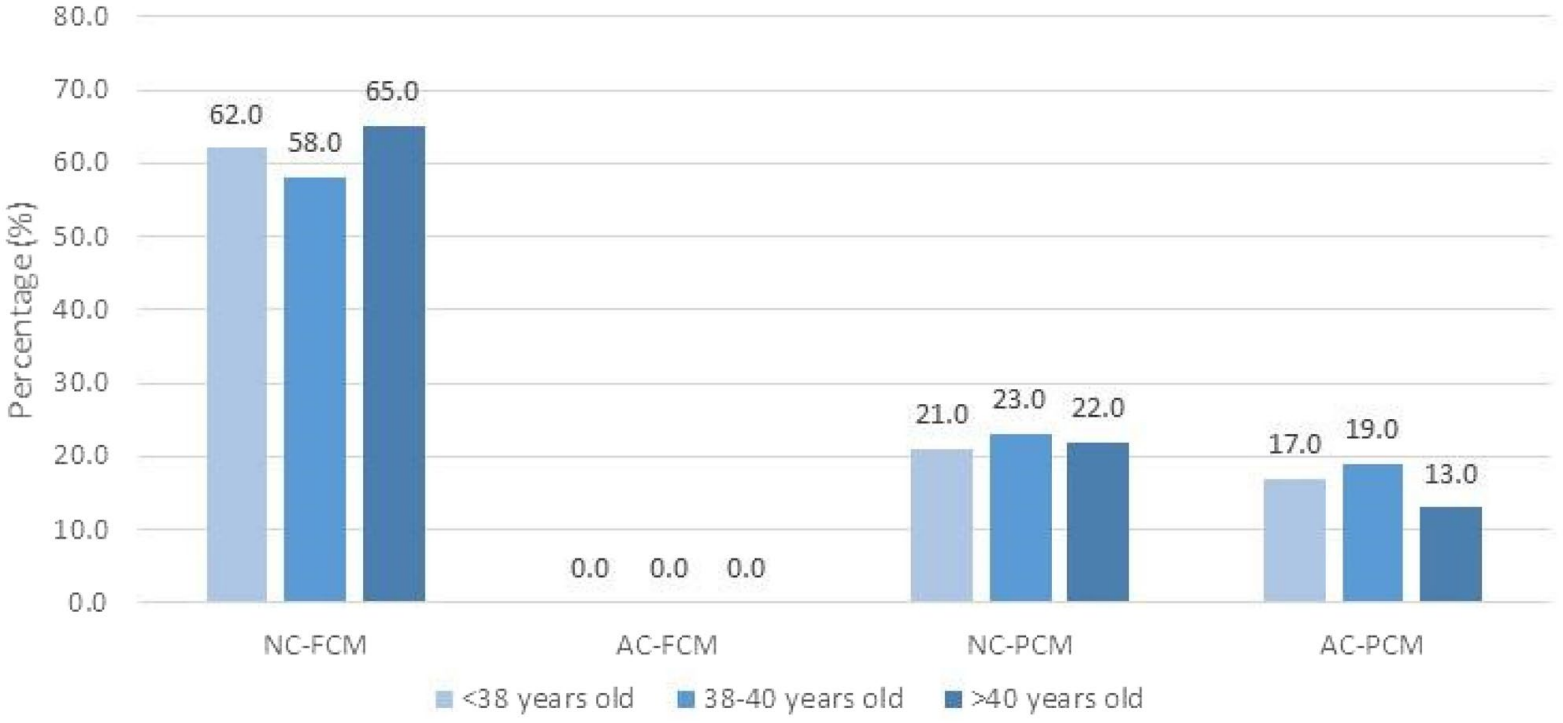
Percentage of embryos according to subgroups of initial zygote cleavage and morula compaction (NC-FCM, NC-PCM, AC-PCM, and AC-PCM), and stratified by female age. Qui-square test. p = 0.500. *NC-FCM* normal cleavage and fully compacted morula, *AC-FCM* abnormal cleavage and fully compacted morula, *NC-PCM* normal cleavage and partially compacted morula, *AC-PCM* abnormal cleavage and partially compacted morula.

Due to the absence of substantial differences between RC and DUC-2 according to female age, they were grouped and called anomalous cleavage (AC). Additionally, the Exc-PCM and Ext-PCM subgroups were merged into a single PCM subgroup, due to the same reason. Then, the embryos were split into four groups according to the initial cleavage pattern (NC or AC) and morula compaction (FCM or PCM) as follows: (i) normal cleavage and fully compacted morulae (NC-FCM); (ii) anomalous cleavage and fully compacted morulae (AC-FCM); (iii) normal cleavage and partially compacted morulae (NC-PCM); and (iv) anomalous cleavage and partially compacted morulae (AC-PCM). Figure 1 shows the distribution of embryos in those four groups according to the female age groups. It is important to note that most embryos with NC resulted in FCM, while no embryos with AC resulted in FCM. There are no differences among the percentages of embryos in each group according to the female age groups.

### Genetic outcomes

**Figure 2:**
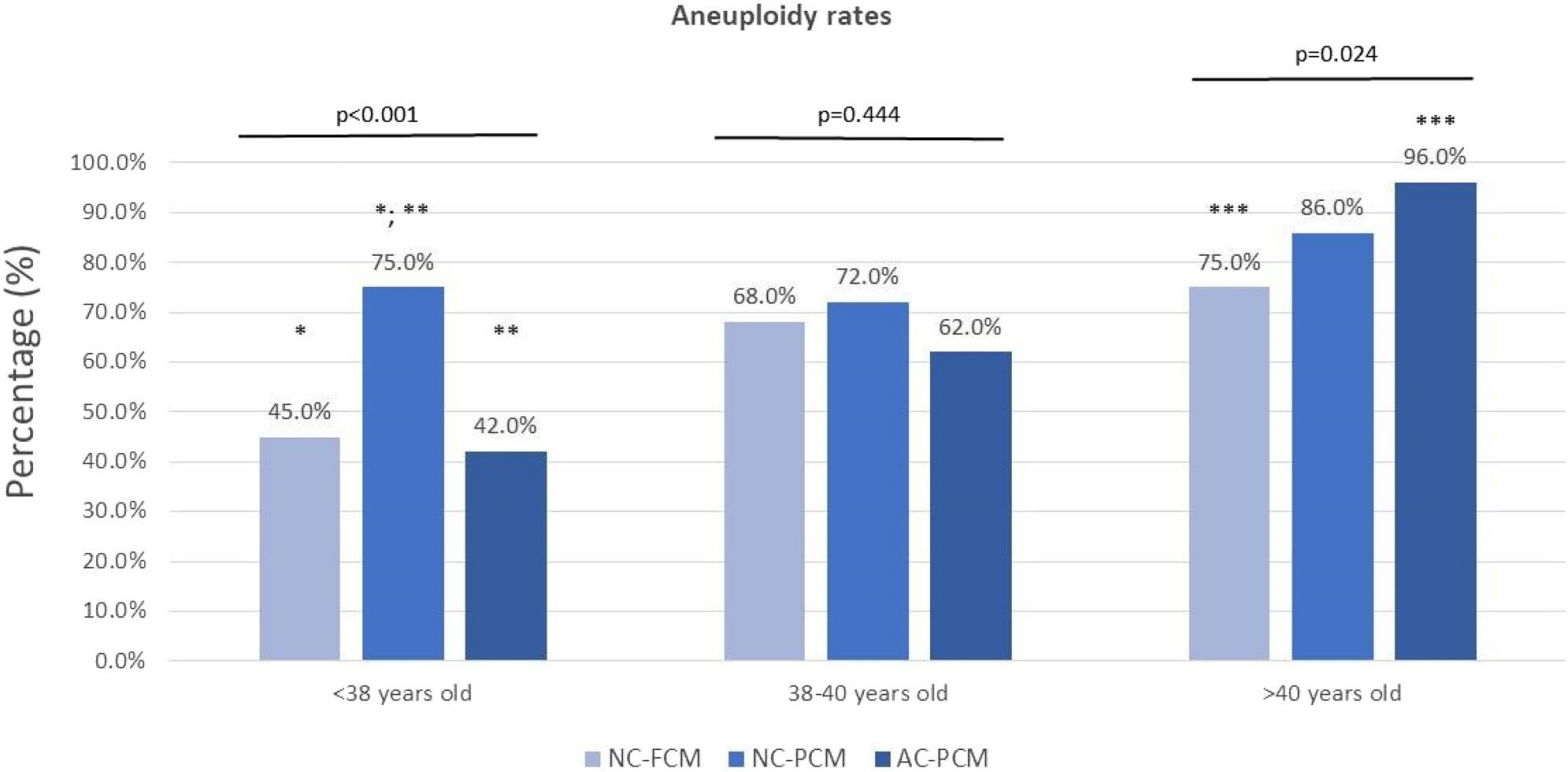
Aneuploidy rates of embryos classified according to subgroups of initial zygote cleavage and morula compaction (NC-FCM, NC-PCM, and AC-PCM), and stratified into female age groups. *NC-FCM* normal cleavage and fully compacted morula, *NC-PCM* normal cleavage and partially compacted morula, *AC-PCM* abnormal cleavage and partially compacted morula. Qui-square test: *p < 0.001; **p < 0.001; ***p = 0.017.

All embryos included in the study underwent biopsy and PGT-A analyses. The aneuploidy rates were evaluated in the three groups of embryos: NC-FCM, NC-PCM, and AC-PCM, as shown in Fig. 2. For women between 38 and 40 years old, the aneuploidy rates were a little higher than < 38 years old and even higher for women > 40 years old, as expected. It is worth mentioning that the embryos of young women (< 38 and 38–40-year-old group) had similar aneuploidy rates for the NC-FCM and AC-PCM embryos. This finding is in agreement with a possible embryo correction mechanism when an error occurs in the first or second mitotic division. On the other hand, it is interesting to note that a high aneuploidy rate was observed for embryos who had normal cleavage but a partially compacted morula (NC-PCM), despite we had observed a statistically significant difference only in women < 38, but not in 38–40-year-old old group.

For women > 40 years old, normally cleaved embryos with a fully compacted morula (NC-FCM) showed lower aneuploidy rates, close to those observed in embryos from women aged 38–40 years. However, the aneuploidy rate was higher in NC-PCM and AC-PCM embryos, suggesting that the previously mentioned putative correction mechanism was not efficient for older women.

### Embryo quality

**Figure 3:**
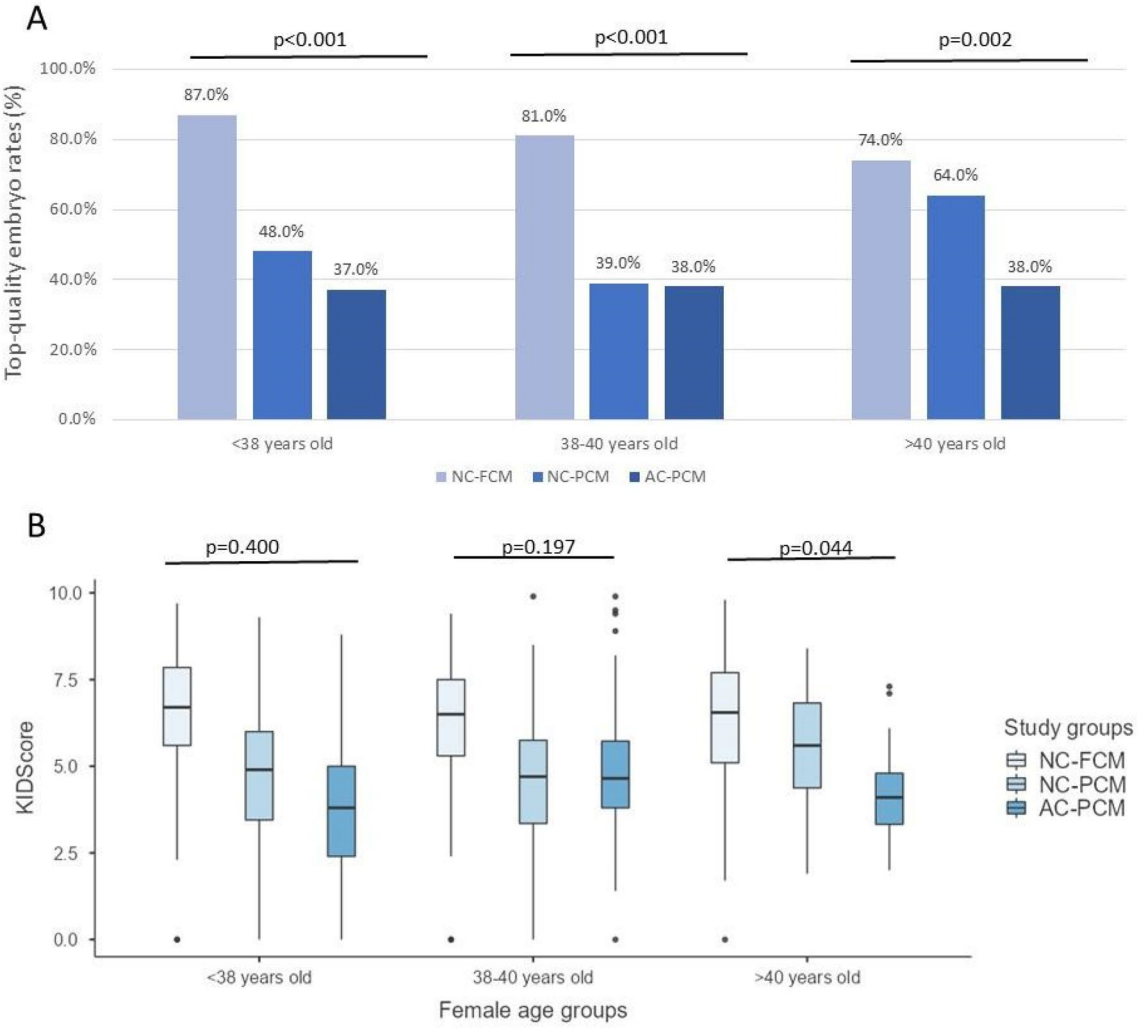
(**A**) Morphological evaluation represented by the top-quality blastocyst rates. (**B**) Morphokinetics evaluation represented by the means of KIDScore and 95% confidence interval. Results were presented according to subgroups of initial zygote cleavage and morula compaction (NC-FCM, NC-PCM, and AC-PCM), and stratified into female age groups. *NC-FCM* normal cleavage and fully compacted morula, *NC-PCM* normal cleavage and partially compacted morula, *AC-PCM* abnormal cleavage and partially compacted morula.

Embryos were evaluated for both morphology and morphokinetics. Regarding morphological parameters, the embryos were classified as top quality (TQ) based on size (grade > 3), ICM, and TE (grades A or B) observed at the blastocyst stage. Morphokinetics were scored using the Embryoscope scoring system, called KIDScore, which is a dynamic embryo assessment based on the time needed to reach each stage of development. Figure 3A shows the percentages of embryos classified as TQ and Fig. 3B shows the average embryo KIDScore in the three groups and according to female age groups. Considering the NC-FCM as the standard group, with no alteration in the early cleavage or compaction process, the percentage of TQ embryos significantly decreased in embryos of NC-PCM and further decreased in AC-PCM for all women ages. The morphokinetic score (KIDScore) averages followed the same pattern as the morphology assessment, however only women > 40 years old had a statistical significance in KIDscore evaluation.

## Discussion

Since the introduction of ovarian stimulation in the early days of IVF, embryo selection has become a crucial task owing to the large number of embryos generated. Consequently, some selection criteria were adopted to discriminate embryos with the highest implantation potential and, ultimately, to improve the probability of pregnancy and live birth^38,39^. A step forward in the predictors of embryo developmental competence, TLS allows non-invasive visualization of time points and aspects of embryonic morphokinetics. Based on these morphokinetic parameters, several embryo selection models have been developed^17,19,40–43^, but despite the staggering amount of data generated, its ability to improve ICSI/IVF outcomes has yielded inconclusive results^44–47^. On the other hand, TLS allows us to observe a series of patterns that were not possible through daily observations. In our study, we analyzed anomalies during early cleavages which can be tracked with the TLS, such as direct cleavage and precocious cleavage. Then, we evaluated how these mitotic anomalies relate to the PCM phenomenon (non-compacted morulae), another condition only possibly observed when embryos are cultured in TLS. Finally, the association of those phenomena with the genetic status of embryos were analysed.

Our data shows that less than 20% of the embryos showed anomalous early cleavage, DUC-2 or RC. However, it is important to consider that, in our study, we only analyzed embryos that reached the blastocyst stage and were biopsied. Thus, several embryos that did not reach the blastocyst stage may have suffered anomalous early cleavage and were therefore not considered here. If the total cohort of zygotes had been analyzed, the proportion of embryos with anomalous early cleavage would likely have been higher.

Moreover, we observed an overall incidence of 38% of PCM among the blastocysts analyzed, slightly lower than previous studies in the literature, such as 55.9% reported by Lagalla et al*.*^37^. When the subgroups were established based on early cleavage and morula compaction (NC-FCM, AC-FCM, NC-PCM, and AC-PCM), all embryos presenting AC, gave rise to a morula with PCM. Moreover, when the aneuploidy rates were compared, we detected that they were similar between the NC-FCM and AC-PCM embryos, at least for women until 40 years old. That is, embryos that had anomalous early cleavage, but excluded or extruded cells during compaction, possibly underwent a “correction mechanism” and thereby eliminated affected blastomeres, which resulted in the same rate of aneuploidy as NC-FCM. This fact supports the hypothesis that PCM is a self-correcting mechanism in embryos that undergo anomalous early cleavage, corroborating other author's findings^35–37^. However, we can't exclude the possibility that embryos with abnormal initial cleavages didn't reach the blastocyst stage and weren't included in this study, since we only evaluated embryos that reached the blastocyst stage and were biopsied.

On the other hand, we observed a subgroup of embryos that did not present visible anomalies at time-lapse but presented a partially compacted morula (NC-PCM). Interestingly, this subgroup showed a significantly higher aneuploidy, mainly in women younger than 38 years. It is possible that this kind of embryo suffered errors in the chromosome segregation process which are not likely to be observed with the time-lapse system visually^48–51^.

Actually, aneuploidy is a common event in human embryos, whether of meiotic or mitotic origin^52^. Meiotic aneuploidy arises mainly from the egg and increases dramatically with maternal age^53^. Mitotic aneuploidy often arises during the first cleavage of the embryo, regardless of maternal age^48,54,55^, as we also observed in our data. Then, the higher prevalence of mitotic errors in older women is justified by the increasing rates of meiotic origin aneuploidy, independently of the presence of mitotic aneuploidy. It is also known that oocytes from older women are less competent^53^, explaining the poorer performance of the correction mechanism.

One detail that deserves our attention is that, despite having similar aneuploidy rates, embryos with AC and/or PCM had morphological grades and KIDScores significantly lower than those of NC-FCM group. A possible explanation for the decrease in morphokinetic and morphological scores is probably the longer development time and poor morphology of these embryos, due to truncated cell divisions and cell exclusion/extrusion processes.

It is known that disturbances in the initial cleavage and compaction processes result in blastomeres asymmetry during embryonic development, which in turn leads to poorer morphological classification. In addition, embryos that show early cleavage anomalies and are supposed to undergo correction mechanisms and may take longer to reach the blastocyst stage. Since the morphokinetic score is mainly based on the times to reach each developmental stage during embryo development, these processes may delay the estimated times, which justifies the differences found regarding the morphokinetic scores in the current study.

In our study, we evaluated only embryos that reached the blastocyst stage with enough quality to be biopsied. This is a limitation as the association between anomalies in the first and second mitotic cleavages and embryonic development arrest could not be evaluated. Another possible limitation is the pooling of different cleavage patterns, which did not allow us to evaluate the cleavage pattern with the efficacy of the supposed correction mechanism in the PCM. However, the study aimed to evaluate the mitotic errors in general, which occur during early cleavage and possibly to be observed in a TLS review. The design of this study allows us to observe the correlations proposed, assuming its weakness. Moreover, the early cleavage anomalies among embryos that reach the blastocyst stage are not common (less than 20% of embryos included in this study) and the subgroup could result in weak statistical analysis. Still, we can consider a weakness of this study, the fact that the observations of the initial mitosis errors were visually evaluated by an embryologist, and subject to observation biases. However, all videos were double-checked by the same senior embryologist in order to minimize classification bias (HM).

The similar aneuploidy rates between the NC-FCM and AC-PCM groups suggest that anomalous cleavage in the first mitotic divisions may give rise to nullisomic blastomeres that do not participate in the morula compaction process and are “eliminated”, not influencing blastocyst euploidy^31^. However, these blastomeres removed from the compaction process confer an inferior morphological appearance and hinder the morphokinetic assessment of the blastocyst that contains them. Therefore, both morphological and morphokinetic assessment should take these embryonic development phenomena into account.

We analyzed live birth rates considering the transfer of a single embryo or transfers of double embryos in which both embryos were in the same classification and/or had the same outcome (implanted or not). Among the data included in this study, we identified 161 euploid embryos transferred in 137 transferred cycles. Live birth rates were compared for NC-FCM 37.4% (n = 115), NC-PCM 30.4% (n = 23) and AC-PCM 34.8% (n = 23) and no statistically significant difference was observed. However, it is important to note that the number of embryos transferred that were classified as NC-FCM was much higher than the other groups, as embryos without abnormal cleavage disorders or morula compaction receive better morphological or morphokinetic classifications, and are primarily selected for transfers, despite all being euploid.

In summary, IVF embryos have been analyzed through the morphology of cleavage-stage embryos and blastocysts, while morula has received little attention. The findings of this study can contribute to selecting embryos for biopsy or transfer, even when an abnormal cleavage is observed, but possibly corrected by PCM.

## Methods

This is a retrospective cohort study based on a databank of anonymized data regarding embryos generated by In Vitro Fertilization (IVF) and cultures in a time-lapse system. All data included in this study refer to couples who underwent IVF cycles with freeze-only embryos, performed as part of routine care in a single assisted reproductive center. All data included referred to cycles with ovarian stimulation and oocyte retrieval, oocyte fertilization using ICSI, embryo culture in a time-lapse system, and blastocyst biopsy for PGT-A. Written informed consent was obtained from all patients before treatment, consenting for clinical and laboratory procedures, embryo biopsy, PGT-A analysis, and the anonymous use of clinical data for research purposes. The project was evaluated and discussed by the internal scientific committee of the Monteleone Assisted Reproduction Center, São Paulo—Brazil. According to Brazilian General Law of Data Protection (Lei nº 13.709, de 14 de agosto de 2018—https://www.in.gov.br/materia/-/asset_publisher/Kujrw0TZC2Mb/content/id/36849373/do1-2018-08-15-lei-no-13-709-de-14-de-agosto-de-2018-36849337), it is exempt from approval by an external Institutional Review Board or specific consenting term, once only anonymized retrospective data were used, and previously consented by patients.

### Data collection

Patients who attended Monteleone Assisted Reproduction Center, São Paulo, Brazil, received a routine treatment according to medical indications and respected the international guidelines and local regulations for assisted reproduction treatments. The clinical and laboratory procedures follow the guidelines established by American Society for Reproductive Medicine (ASRM Practice Committee, https://www.asrm.org/news-and-publications/practice-committee-documents/), European Society for Human Reproduction and Embryology (ESHRE Guidelines, Consensus and recommendations, https://www.eshre.eu/Guidelines-and-Legal), Brazilian Association for Assisted Reproduction (*Associação Brasileira de Reprodução Assistida*—SBRA, https://sbra.com.br/publicacoes-nova/), and Federal Counsel of Medicine (*Conselho Federal de Medicina*—CFM https://sistemas.cfm.org.br/normas/visualizar/resolucoes/BR/2022/2320).

For this study, only data from embryos biopsied for genetic analysis were included. The database included 835 embryos from 195 ICSI cycles performed from April 2018 to June 2020 at Monteleone Assisted Reproduction Center, São Paulo, Brazil. The inclusion criteria were data from IVF cycles in which: (i) embryos were individually cultured and monitored from the zygote to the blastocyst stage in a time-lapse incubator (EmbryoScope Plus, Vitrolife, Midtjylland, Denmark), (ii) Blastocysts were analyzed using next-generation sequencing (NGS) after a single trophectoderm biopsy. Among 195 ICSI cycles included, couples indications for IVF treatment were mostly advanced maternal age (≥ 38 years, 66.7%) correlated with other factors. Unexplained infertility (7.3%), male factor (6.8%), and other factors (19.2%) were responsible for the remaining cases. For all cycles, women underwent controlled ovarian stimulation, oocyte recovery, and MII oocytes were fertilized by ICSI.

As routine, prospectively data are annotated in time-lapse system software (Embryoscope, Vitrolife). For this study, retrospective reanalysis of the time-lapse videos was made by an expert embryologist for the presence of abnormalities at early cleavage and compaction stages. All video analyses were performed by the same embryologist (HM). Normal early cleavage was defined by the first cleavage of one blastomere to two daughter blastomeres, and then the second cleavage where either daughter blastomere cleaved again, resulting in four blastomeres. DUC occurring at second cleavage (DUC-2), resulting in five blastomeres, and RC, which occurs shortly after the first mitotic division when one of the daughter blastomeres divides into two or more blastomeres in less than 5 h, were recorded.

Morulae were classified as fully compacted morulae (FCM, n = 516), when no excluded/extruded cells were observed, or partially compacted morulae (PCM) showing cells excluded/excluded from the compaction process. The PCM was further subdivided into other two groups, PCM showing excluded cells (Exc-PCM), and PCM showing extruded from an already-compacted morula, added of PCM showing both patterns, excluded and extruded cells (Ext-PCM).

Embryo morphology and developmental dynamics were noninvasively observed by capturing images with 11 focal planes at 10-min intervals. The timing of pronuclei fading, to two, three, four, five, eight and nine cells (t2, t3, t4, t5, t8, and t9, respectively), the morula (tM), and blastulation (tB) were recorded. The KIDScoreD5 v2 was used to embryo morphokinetics classification. Moreover, morphology classification of blastocysts were performed and blastocyst expansion, inner cell mass (ICM) and trophectoderm (TE) epithelium quality were graded according to the Gardner scoring system^56^.

### Statistical analysis

The parameters of initial zygote cleavage and morula compaction were evaluated and correlated with the genetic, morphokinetics, and morphological embryonic outcomes. Quantitative variables were compared using Student’s *t* test or analysis of variance, and Chi-squared or Fisher exact test analysis was performed for the comparison of categorical data. Jamovi 2.3.13 statistical package was used and differences were considered statistically significant at p < 0.05.

## Data Availability

The datasets used and/or analyzed during the current study are available from the corresponding author on request.

## References

[1] Zaninovic N, Irani M, Meseguer M (2017). Assessment of embryo morphology and developmental dynamics by time-lapse microscopy: Is there a relation to implantation and ploidy?. Fertil. Steril..

[2] Fragouli E, Alfarawati S, Spath K, Wells D (2014). Morphological and cytogenetic assessment of cleavage and blastocyst stage embryos. Mol. Hum. Reprod..

[3] Rienzi L (2019). Time of morulation and trophectoderm quality are predictors of a live birth after euploid blastocyst transfer: A multicenter study. Fertil. Steril..

[4] Capalbo A (2014). Correlation between standard blastocyst morphology, euploidy and implantation: An observational study in two centers involving 956 screened blastocysts. Hum. Reprod..

[5] Alfarawati S (2011). The relationship between blastocyst morphology, chromosomal abnormality, and embryo gender. Fertil. Steril..

[6] Munné S (2019). Preimplantation genetic testing for aneuploidy versus morphology as selection criteria for single frozen-thawed embryo transfer in good-prognosis patients: A multicenter randomized clinical trial. Fertil. Steril..

[7] Yang Z (2012). Selection of single blastocysts for fresh transfer via standard morphology assessment alone and with array CGH for good prognosis IVF patients: Results from a randomized pilot study. Mol. Cytogenet..

[8] Scott RT (2013). Blastocyst biopsy with comprehensive chromosome screening and fresh embryo transfer significantly increases in vitro fertilization implantation and delivery rates: A randomized controlled trial. Fertil. Steril..

[9] Rosenwaks Z (2018). The pros and cons of preimplantation genetic testing for aneuploidy: Clinical and laboratory perspectives. Fertil. Steril..

[10] Desai N, Goldberg JM, Austin C, Falcone T (2018). Are cleavage anomalies, multinucleation, or specific cell cycle kinetics observed with time-lapse imaging predictive of embryo developmental capacity or ploidy?. Fertil. Steril..

[11] Gleicher N (2017). A single trophectoderm biopsy at blastocyst stage is mathematically unable to determine embryo ploidy accurately enough for clinical use. Reprod. Biol. Endocrinol..

[12] Rubio C, Racowsky C, Barad DH, Scott RT, Simon C (2021). Noninvasive preimplantation genetic testing for aneuploidy in spent culture medium as a substitute for trophectoderm biopsy. Fertil. Steril..

[13] Coates A (2017). Optimal euploid embryo transfer strategy, fresh versus frozen, after preimplantation genetic screening with next generation sequencing: A randomized controlled trial. Fertil. Steril..

[14] Simon AL (2018). Pregnancy outcomes from more than 1,800 in vitro fertilization cycles with the use of 24-chromosome single-nucleotide polymorphism-based preimplantation genetic testing for aneuploidy. Fertil. Steril..

[15] Conaghan J (2013). Improving embryo selection using a computer-automated time-lapse image analysis test plus day 3 morphology: Results from a prospective multicenter trial. Fertil. Steril..

[16] Herrero J, Meseguer M (2013). Selection of high potential embryos using time-lapse imaging: The era of morphokinetics. Fertil. Steril..

[17] Kirkegaard K (2014). Limitations of a time-lapse blastocyst prediction model: A large multicentre outcome analysis. Reprod. Biomed. Online.

[18] Apter S (2020). Good practice recommendations for the use of time-lapse technology. Hum. Reprod. Open.

[19] Meseguer M (2011). The use of morphokinetics as a predictor of embryo implantation. Hum. Reprod..

[20] Desai N (2014). Analysis of embryo morphokinetics, multinucleation and cleavage anomalies using continuous time-lapse monitoring in blastocyst transfer cycles. Reprod. Biol. Endocrinol..

[21] Milewski R, Czerniecki J, Kuczyńska A, Stankiewicz B, Kuczyński W (2016). Morphokinetic parameters as a source of information concerning embryo developmental and implantation potential. Ginekol. Pol..

[22] McCoy RC (2018). Tripolar chromosome segregation drives the association between maternal genotype at variants spanning PLK4 and aneuploidy in human preimplantation embryos. Hum. Mol. Genet..

[23] Ottolini CS (2017). Tripolar mitosis and partitioning of the genome arrests human preimplantation development in vitro. Sci. Rep..

[24] Meriano J, Clark C, Cadesky K, Laskin CA (2004). Binucleated and micronucleated blastomeres in embryos derived from human assisted reproduction cycles. Reprod. Biomed. Online.

[25] Zhan Q, Ye Z, Clarke R, Rosenwaks Z, Zaninovic N (2016). Direct unequal cleavages: Embryo developmental competence, genetic constitution and clinical outcome. PLoS One.

[26] Ozbek IY (2021). Comparison of single euploid blastocyst transfer cycle outcome derived from embryos with normal or abnormal cleavage patterns. Reprod. Biomed. Online.

[27] Ciray HN (2014). Proposed guidelines on the nomenclature and annotation of dynamic human embryo monitoring by a time-lapse user group. Hum. Reprod..

[28] Rubio I (2012). Limited implantation success of direct-cleaved human zygotes: A time-lapse study. Fertil. Steril..

[29] Kalatova B, Jesenska R, Hlinka D, Dudas M (2015). Tripolar mitosis in human cells and embryos: Occurrence, pathophysiology and medical implications. Acta Histochem..

[30] Yang ST (2015). Cleavage pattern predicts developmental potential of day 3 human embryos produced by IVF. Reprod. Biomed. Online.

[31] McCollin A, Swann RL, Summers MC, Handyside AH, Ottolini CS (2020). Abnormal cleavage and developmental arrest of human preimplantation embryos in vitro. Eur. J. Med. Genet..

[32] Harada Y (2020). Selection of high-quality and viable blastocysts based on timing of morula compaction and blastocyst formation. Reprod. Med. Biol..

[33] Coticchio G, Lagalla C, Sturmey R, Pennetta F, Borini A (2019). The enigmatic morula: Mechanisms of development, cell fate determination, self-correction and implications for ART. Hum. Reprod. Update.

[34] Mayer RB (2018). Good-quality blastocysts derived from vacuolized morulas show reduced viability. Fertil. Steril..

[35] Coticchio G (2021). Perturbations of morphogenesis at the compaction stage affect blastocyst implantation and live birth rates. Hum. Reprod..

[36] Lagalla C (2017). Embryos with morphokinetic abnormalities may develop into euploid blastocysts. Reprod. Biomed. Online.

[37] Lagalla C (2020). Alternative patterns of partial embryo compaction: Prevalence, morphokinetic history and possible implications. Reprod. Biomed. Online.

[38] Ebner T, Moser M, Sommergruber M, Tews G (2003). Selection based on morphological assessment of oocytes and embryos at different stages of preimplantation development: A review. Hum. Reprod. Update.

[39] Scott L, Finn A, O'Leary T, McLellan S, Hill J (2007). Morphologic parameters of early cleavage-stage embryos that correlate with fetal development and delivery: Prospective and applied data for increased pregnancy rates. Hum. Reprod..

[40] Basile N (2015). The use of morphokinetics as a predictor of implantation: A multicentric study to define and validate an algorithm for embryo selection. Hum. Reprod..

[41] Motato Y (2016). Morphokinetic analysis and embryonic prediction for blastocyst formation through an integrated time-lapse system. Fertil. Steril..

[42] Campbell A (2013). Modelling a risk classification of aneuploidy in human embryos using non-invasive morphokinetics. Reprod. Biomed. Online.

[43] Milewski R (2015). A predictive model for blastocyst formation based on morphokinetic parameters in time-lapse monitoring of embryo development. J. Assist. Reprod. Genet..

[44] Armstrong S (2019). Time-lapse systems for embryo incubation and assessment in assisted reproduction. Cochrane Database Syst. Rev..

[45] Bhide P (2019). Time lapse imaging of embryos is useful in in vitro fertilisation (IVF) or intracytoplasmic sperm injection (ICSI) treatment: AGAINST: The jury is still out. BJOG.

[46] Gallego RD, Remohí J, Meseguer M (2019). Time-lapse imaging: The state of the art†. Biol. Reprod..

[47] Bhide P (2020). TILT: Time-Lapse Imaging Trial-a pragmatic, multi-centre, three-arm randomised controlled trial to assess the clinical effectiveness and safety of time-lapse imaging in in vitro fertilisation treatment. Trials.

[48] Cavazza T (2021). Parental genome unification is highly error-prone in mammalian embryos. Cell.

[49] Brooks KE (2022). Molecular contribution to embryonic aneuploidy and karyotypic complexity in initial cleavage divisions of mammalian development. Development.

[50] Currie CE (2022). The first mitotic division of human embryos is highly error prone. Nat. Commun..

[51] Palmerola KL (2022). Replication stress impairs chromosome segregation and preimplantation development in human embryos. Cell.

[52] Vanneste E (2009). Chromosome instability is common in human cleavage-stage embryos. Nat. Med..

[53] Gruhn JR (2019). Chromosome errors in human eggs shape natural fertility over reproductive life span. Science.

[54] Daughtry BL, Chavez SL (2016). Chromosomal instability in mammalian pre-implantation embryos: Potential causes, detection methods, and clinical consequences. Cell Tissue Res..

[55] Chavez SL (2012). Dynamic blastomere behaviour reflects human embryo ploidy by the four-cell stage. Nat. Commun..

[56] Gardner DK, Lane M, Stevens J, Schlenker T, Schoolcraft WB (2000). Blastocyst score affects implantation and pregnancy outcome: Towards a single blastocyst transfer. Fertil. Steril..

